# Comparison of Different Nutritional Screening Approaches and the Determinants of Malnutrition in Under-Five Children in a Marginalized District of Punjab Province, Pakistan

**DOI:** 10.3390/children9071096

**Published:** 2022-07-21

**Authors:** Muhammad Shahid, Yongshuan Liu, Waqar Ameer, Madeeha Gohar Qureshi, Farooq Ahmed, Kun Tang

**Affiliations:** 1 Vanke School of Public Health, Tsinghua University, Beijing 100084, China; de202159006@uibe.edu.cn; 2 School of Insurance and Economics, University of International Business and Economics (UIBE), Beijing 100029, China; 3 Party Committee Office, University of International Business and Economics (UIBE), Beijing 100029, China; liuyongshuan@uibe.edu.cn; 4 Department of Economics, Shandong Business and Technology University, Yantai 264005, China; waqar.ameer@yahoo.com; 5 Department of Economics, Pakistan Institute of Development Economics, Islamabad 44000, Pakistan; madeeha.qureshi@pide.org.pk; 6 Department of Anthropology, Quaid-i-Azam University, Islamabad 44400, Pakistan

**Keywords:** malnutrition, body mass index, composite index of anthropometric failure, mid-upper arm circumstances, wasting, underweight, stunting, Pakistan

## Abstract

Objectives: This research measures the occurrence of malnutrition amongst under-five children in the Rahimyar Khan district of Southern Punjab in Pakistan. Employing different anthropometric measurement approaches such as (1) conventional indices (HAZ, WAZ, and WHZ), (2) CIAF, (3) BMI-for-age, and (4) MUAC, we compare their estimated results and examine the relationship between socioeconomic determinants and different anthropometric indicators. Methods: The study employs a proportional purposive random sampling method to collect data from 384 rural households in the community-based study using a self-administered survey and following the Lady Health Workers (LHWs) registered records. The nutritional status of 517 under-five children is measured with references to WHO (2009) child growth standards. Furthermore, the investigation used the model of binary logistic regression to measure the impact of socioeconomic factors on child malnutrition. Results: Compared with other approaches, the CIAF identifies more malnourished children (63%). The results of binary logistic regression illustrate that all the explanatory variables indicate a more significant empirical association with CIAF than conventional indices, BMI-for-age, and MUAC. Conclusion: CIAF is a more reliable tool for assessing child nutrition because it not only demonstrates more accurate estimates of malnutrition but also recognizes children with multiple anthropometric failures.

## 1. Introduction

Globally, 19% (110 million) of children below the age of five are severely underweight, while 30% (170 million) are stunted [1]. About 2/3 of malnourished children live in Asia [2,3]. Pakistan Demographic and Health Survey (2017–2018) showed that stunting occurrence is 38%, underweight is 23%, and wasting is 8%. Aimed at measuring the nutritional status of children, conventional anthropometric indexes, for example, underweight (WAZ), wasting (WHZ), and stunting (HAZ), have been extensively utilized. According to WHO, these indices reflect distinguishable biological processes: wasting as acute malnutrition; stunting as chronic malnutrition; underweight as a mixture of chronic and acute malnutrition having no distinction [4]. These three catalogs signify different features of malnutrition. Nonetheless, they typically coincide and are not commonly exclusive [5]. Therefore, conventional indices have been debated to be insufficient for evaluating the general occurrence rate of malnutrition among younger children. To control this inadequacy, propositions were given for developing a new aggregate of indicators [6,7].

Svedberg [8] proposed the CIAF that offers distinct figures in a population for the whole estimation of malnourished children. More specifically, its multiple subdivisions of anthropometric failures can forecast the possibility of ailment and mortality. These features distinguish it from the other current indicators that cannot estimate separately with high precision [9,10]. Even though CIAF was considered a valuable compound scale, it neglects singular commitment, significance of hindering, underweight, and wasting compared with the general commonness of malnutrition. In the 19th century, Adolphe Quetelet created a get-up-to-speed approach or BMI-for-age, which estimates the nourishing status of grown-ups. During the 1970s, researchers considered BMI a good proxy for adipose and overweight-related problems [11]. Catch-up growth can aid in recognizing which youngsters are disposed to catch up and which are at threat of falling further or staying behind. The WHO and UNICEF’s joint statement was issued regarding WHO’s growth standards in May 2009 to identify severe malnourishment in newborns and children, and a new measurement tool, MUAC tape, was made available [11,12].

Several studies have comparatively analyzed and discussed the different anthropometric measurement approaches. Most analyze whether the conventional indices or CIAF is a better approach to screening more malnourished children. However, limited studies have discussed comparing more than two anthropometric measurement tools for malnutrition screening. To the knowledge of the present investigation, no such investigation from Pakistan has evaluated the malnourishment screening tools or comparison of malnutrition prevalence through different approaches. Most of the literature in Pakistan is on socioeconomic determinants of malnutrition or the incidence of malnutrition. Therefore, the current study not only measures the occurrence of malnourishment in under-five children but also draws a comparison among them for screening malnutrition among children in one of the marginalized and high malnutrition prevalence districts of Punjab. Furthermore, this research investigates the association between socioeconomic determinants and different anthropometric indicators, which is missing in the literature.

## 2. Materials and Methods

### 2.1. Study Area

The district of Rahimyar Khan is located in the South Punjab province in Pakistan. It covers an area of 11,880 km with two main ecological zones-desert and agricultural ground land. It is divided into four administrative sub-districts. According to the 2017 census of Pakistan, the district’s population is 4,814,006, of which ~78% reside in rural areas; 50% are deprived of proper sanitation facilities [13]. Also, it has one of the highest malnutrition prevalence districts among 36 districts in Punjab province (MICS-2014). Almost 77% of mothers are illiterate; 91% of the households are deprived of basic amenities of life, and around about 58% of the families have yearly earnings of less than 50,000 PKR. However, 26% of families’ yearly earnings are less than 100,000 PKR.

### 2.2. Sampling and Research Design

The current investigation was a community-based and cross-sectional exploration, and 384 rural households were part of the primary data collection by a self-applied survey using a balanced purposive random sampling method. Keeping a 5% confidence interval and 95% confidence level, the data were allocated proportionally surrounded by four sub-districts: (1) Khan Pur, (2) Rahimyar Khan, (3) Liaquatpur, and (4) Sadiqabad. The statistical strategy focused on the probability of size ratios in each of the 4 sub-district. A stratified random sampling of Union Councils (UC) in each sub-district was included in the first phase. The rural families for the study were selected arbitrarily with the help of the record from a female health worker. The sample size (n = 384 families) was derived with the help of the Raosoft calculator by reviewing the 5% confidence interval and 95% confidence level. The calculations of the sample size (n) are described underneath:

n = Z^2^ × (p) × (1 − p)/c^2^

In the above equation:

Z = A value that is 1.96 for 95% confidence level

p = It refers to percentage picking a choice and expressed as decimal (0.5 used for sample size needed)

c = It means confidence interval and expressed as decimal (i.e., 0.04 = ±4)

n = (1.96)^2^ × (0.5) × (1 − 0.5)/(0.05)^2^ = 384.16 = 384

In Table 1 description of the proportional allocation in twelve UCs of n = 384 is mentioned:

NI = n × Ni/N

Formula for each UC sample calculation = Population of UC 1,2,3/total Population of 3 UCs × sample size of tehsil

NI = number of sampled respondents in each UC

i = number of the UCs in the study area, i.e., 1, 2, 3,…,12

N = total size of the sample

### 2.3. Data Collection and Ethical Consideration

Children in a household under five were considered for the survey. If more than one household existed in a single building, or families combined into a single household during the survey, then the investigation took them as independent if they prepared meals separately. The anthropometric assessments were carried out by female health workers, given training by the principal author before allocating the task of anthropometric assessment. The study utilized height assessments tape, weight machine, and MUAC tape to gather data for the measurement of age, weight, and height of the samples.

Research data were collected for three months between November 2017 to January 2018. After getting study approval from the office of the district health officer, mothers of children and their close relatives were brought up-to-date in local languages with the help of female health workers who told them about the type of the investigation one week prior to seek their verbal agreement, as well as readying them to contribute in the investigation. Mothers belonging to 384 different families agreed to contribute willingly to the research, and all 384 mothers provided their verbal agreement in the meeting before the interview. In black and white, consent was not taken as 74% of mothers were uneducated and also showed reluctance because of their cultural boundaries.

The study was approved in the 6th meeting of the Graduate Research Management Council (GRMC) of the Pakistan Institute of Development Economics [No. HE-01/2017 (PIDE)]. GRMC serves as an institutional review board. In addition, the Department of Health Economics at PIDE and the District Health Office in Rahimyar Khan also checked and approved the tools and protocols of this study. Furthermore, all the study details were explained to health officials, LHWs, and mothers before taking their consent.

### 2.4. Measurements, Terminologies, and Variables

Stunting: z-score of height-for-age i.e., (HAZ) < −2 SD was termed as stunting; wasting: z-score of weight-for-height i.e., (WHZ) < −2 SD was termed as wasting; underweight: z-score of weight-for-age i.e., (WAZ) < −2 SD was termed as underweight. The CIAF sees the general occurrence of malnourishment within children. Based on this classification, children are distributed into 7 groups: group-A for “No Failure”, group B for Stunted, group C for Wasted, group D for Underweight, group E for Stunted + Underweight, group F for Wasted + Underweight, group G for Stunted + wasted, and group-H for Stunting + Wasting + Underweight. The total for malnourishment occurrence is measured by combining all groups excluding A. CIAF was dichotomized, “1” for the undernourished child and “0” for a non-undernourished child. CIAF was established on three indexes, such as stunting, underweight, and wasting, specified by World Health Organization (WHO) children development standards rules using anthropometric assessments [11]. The current study considered BMI-for-age z-scores. If BMI-for-age z-scores is < −2 SD, it is defined as undernutrition. Similarly, MUAC tape is in centimeters and marked in colors [red: 0–11.5 cm = SAM; yellow: 11.5–12.5 cm = MAM; and green: ≥ 12.5 cm], was used for the screening undernutrition. According to WHO and UNICEF criteria, MUAC < 12.5 cm is defined as malnutrition, and MUAC ≥ 12.5 cm determines the child is not malnourished [11,12].

The detail about CIAF classification of children with anthropometric failure is given below in Table 2:

The investigation predictor variables were the age of children in months, gender of the child, childbirth order, maternal educational status, maternal employment status, and household socioeconomic status (poor, middle, and rich). To measure the socioeconomic status, this study constructed a simple index of socioeconomic status based on six indicators: (1) mud type house or have cemented type household, (2) family is some landholding or not, (3) facility of electricity is available in the household or not, (4) within the house water drinking facility is present or absent, (5) any family member in the house is educated or not, (6) House keeps Television, newspaper, and radio or not. The response for these six indicators was in binary form (0 or 1). The total scores for the socioeconomic status index were 0 to 6 after adding these six indicators. These six indicators represent the household’s basic life amenities. Those having none or 1 or 2 items are included in the SES-1 category, representing the poor socioeconomic status of the household. The middle socioeconomic status (SES-2 category) of households was indicated when households had 3 to 4 items. Those households having 5 to 6 items were considered to be in the SES-3 category, representing their financial socioeconomic status. Studies in India and Pakistan used the household deprivation status index, which depicts the socioeconomic status (SES). We followed those studies to construct the SES index [14,15,16].

### 2.5. Statistical Analysis

Before constructing the anthropometric indicators, data cleaning was done, and outliers were drawn out. Z-scores that did not fit the WHO flags were reduced from the data set while estimating the CIAF. Of 517 under-five children, 316 were included in the study, and 201 were skipped because of over range (less than −5 and greater than +5). Excel 2013 was used for data entry, while the STATA-15, Excel 2013, and WHO Anthro software were used to conduct the analysis. Descriptive statistics were performed to compute and then compare the rates of malnutrition through different anthropometric approaches. Furthermore, to measure the impact of socioeconomic dynamics on child malnourishment, the binary logistic regression model was used.

The logistic regression method assesses the chances of malnourishment in basically two methods: 1 = if a child is undernourished, or 0 = if a child is not undernourished. The hypothesis set in the present investigation is that the malnourishment status of children is affected by many socioeconomic indicators. Malnutrition in this study was assessed through different anthropometric approaches. So, Child BMI-for-age, CIAF, and conventional indices (stunting, wasting, and underweight) as dependent variables. Similarly, MUAC < 12.5 cm coded 1, which means the child is malnourished, and MUAC ≥ 12.5 cm coded as 0, showing the child is not malnourished. These outcomes or dependent variables were set in binary form (0 or 1). The model logistic regression measures the chance of the dependent variable, which is malnourishment of the child conditioned on socioeconomic signs. The binary logistic regression description form is given below:

P (Yi = 1|X_1i_, X_2i_,…, Xkn) = F (β_0_ + β_1_X_1i_ + β_2_X_2i_ + … + β_n_X_kn_)

In this equation, Y_i_ is denoting the child’s signs of malnourishment “i” as the outcome variable (child BMI-for-age, CIAF, MUAC, stunting, wasting, and underweight); X shows the explanatory variables; the coefficients of interest are represented by β’s, which elucidate the amount of correlation with outcome variables. And Y, a dependent or binary outcome variable, and (Yi = 0) indicates no undernourishment in a child, and (Yi = 1) represents that the child is undernourishment/stunted/underweight/wasted/low BMI/low MUAC, X = (X_1i_, X_2i_,…,Xkn) as predictor variables, and xi is seen as the predictor variable value for “i” observation.

## 3. Results

**Figure 1:**
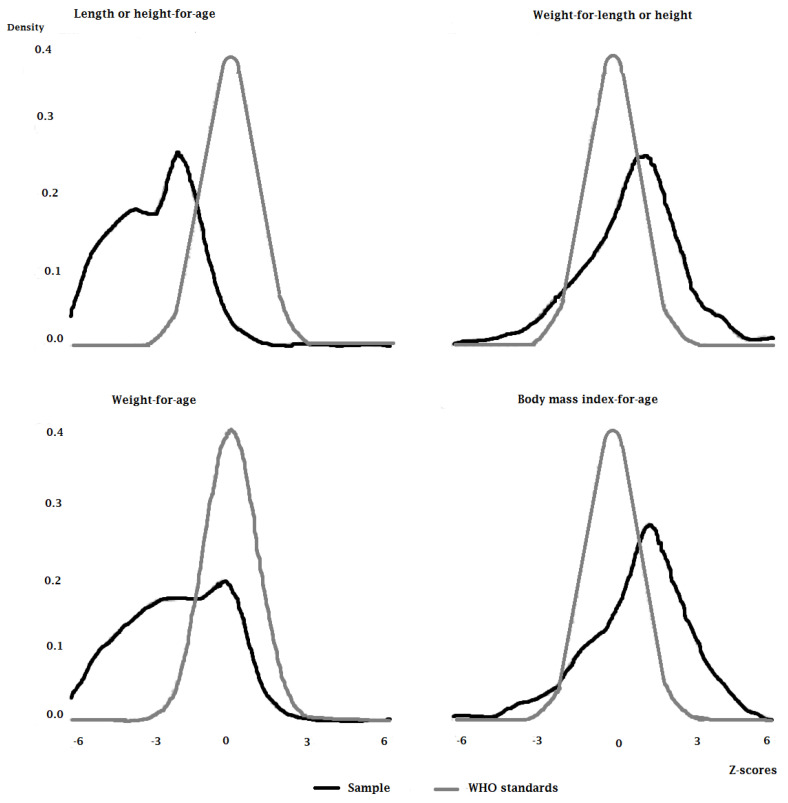
Z−Scores distribution of sample.

**Figure 2:**
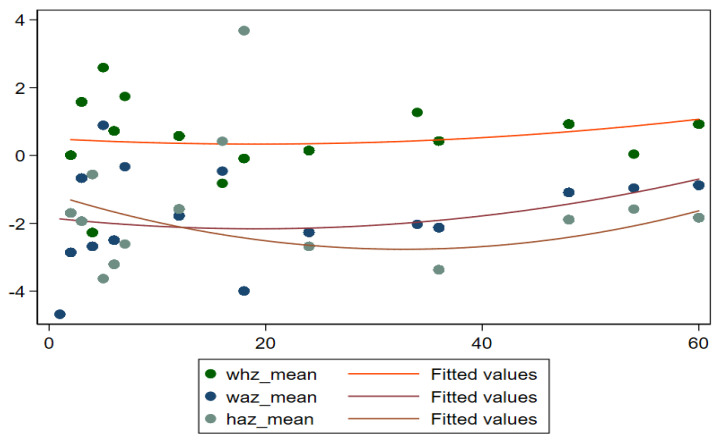
Mean Z−Scores (HAZ, WHZ, and WAZ) by age in months.

Present exploration focused on the nutritive status of 517 under-5 children. Of the total, 55.32% were male and 44.68% were female. After the anthropometric assessment, it is first important to look at the data distribution. In the distribution, <−1 to >−2 z-scores show normal category, <−2 to >−3 shows moderate malnutrition, and <−3 to >−5 or −6 shows serve malnutrition. WHO does not consider the z-scores >−6. Figure 1 and Figure 2 explain that there exist deficiencies in HAZ (stunting) and WAZ (underweight), though a mere precise and limited indication of WHZ (wasting) and BMI-for-age is present.

**Figure 3:**
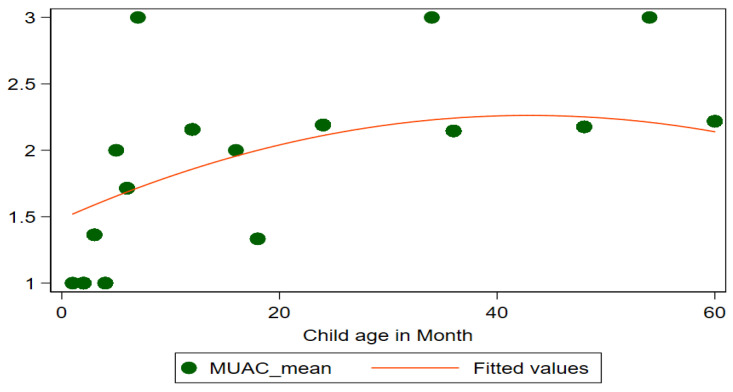
Mean Z−Scores (MUAC) by age in months.

**Figure 4:**
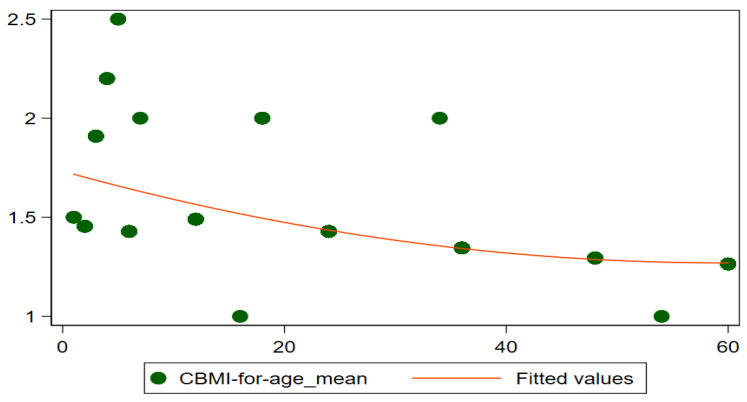
Mean Z−Scores (CBMI) by age in months.

To understand the nature of malnutrition, it can be useful to look at the mean z-scores of children’s ages in months. Figure 2 exposes the details of mean z-scores for different anthropometric indicators such as WAZ (underweight), HAZ (stunting), and WHZ (wasting) in children below five. It shows that mean z-scores for all children of district Rahimyar Khan slightly increase after the age of 40 months. Figure 3 shows that mean z-scores for MUAC for all children increase till age 40 months and then slightly decrease. Furthermore, Figure 4 shows mean z-scores child BMI-for-age decreasing over age.

**Figure 5:**
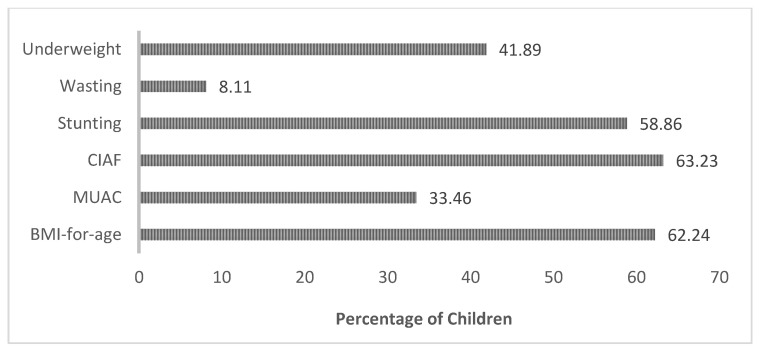
Malnutrition prevalence according to BMI-for-age, MUAC, CIAF, and Conventional Anthropometric Indices (Stunting, Wasting, and Underweight).

The prevalence of malnutrition using different approaches, i.e., BMI approach, MUAC, and conventional anthropometric indices (Stunting, Wasting, and underweight) in children, is given in Figure 5.

The results in Figure 5 demonstrate that BMI identified 62.24% of children are undernourished, CIAF identified 63.23% of children are malnourished, while MUAC depicted that 33.46% of children are malnourished. While according to conventional indices, the underweight occurrence was 41.89%, stunting was 58.86%, and wasting was 8.11% in the Rahimyar Khan district.

Table 3 shows frequencies and percentages of malnutrition prevalence through dif-ferent outcome approaches among preschool children in district Rahimyar Khan. Results depicted in Table 3 show that malnutrition prevalence rates are high. The study investi-gated shocking results through the BMI-for-age approach: 12% of the sample were over-weight while 14% were obese in the Rahimyar Khan district.

**Figure 6:**
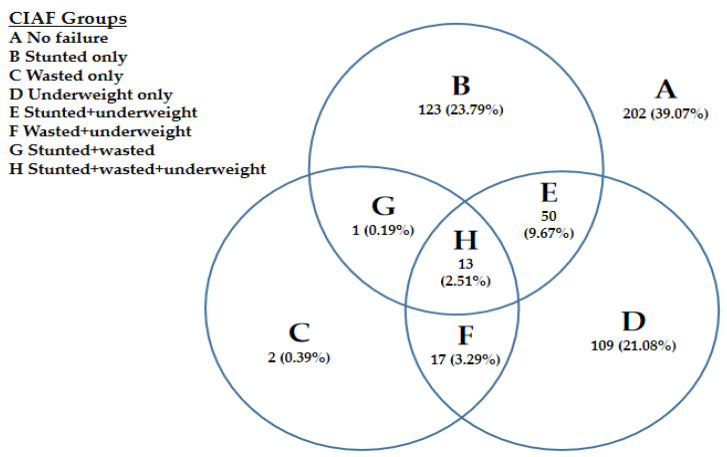
overall malnutrition prevalence (multiple anthropometric failures) according to the Subgroups of CIAF classification.

The results in Figure 6 show the multiple anthropometric failures according to the subgroups of CIAF classification among children. The results showed that stunting prevalence was 23.79%, underweight was 21.08%, and wasting was 0.39% using CIAF classifications. Other anthropometric disorders are also explained by CIAF in Figure 6.

Logistic Regression Estimates

In the current study, it was supposed that child growth is affected by different socioeconomic factors. For this purpose, the study measured the association between child health indicators and with age and sex of the child.

The logistic regression estimates for CIAF and other anthropometric indicators are represented in Table 4. The logistic findings in the CIAF model for children’s age showed that the children’s age was correlated with a greater probability of malnourishment (OR = 6.33, 95% CI: 2.24–17.90). The chances of male child malnourishment were lesser in comparison to female counterparts (OR = 0.69, 95% CI: 0.41–1.16). Children having 4–5 years of birth order contains lesser possibilities of undernourishment (OR = 0.44, 95% CI: 0.21–0.94). Mothers with primary education had lower probabilities of malnourishment in their children under five (OR = 0.002, 95% CI: 1.48–5.48). However, unemployed mothers had a greater likelihood of malnourished children under five (OR = 8.06, 95% CI: 0.93–69.93). Through the scores of SES, the probabilities of children becoming undernourished were less in the SES category 2 families (OR = 0.02, 95% CI: 0.05–0.88), and chances were also less in the SES category 3 families (OR = 0.001, 95% CI: 0.01–0.16).

## 4. Discussion

Conventional indices (HAZ, WAZ, and WHZ), CIAF, BMI-for-age, and MUAC estimated malnutrition in children of preschool age in rural Southern Punjab, Pakistan. The results were as follows: CIAF approach identified 63%, MUAC, 33.46%, BMI-for-age, 62.24%. Conventional indices showed Stunting: 58.86%, Wasting: 8.11%, and Underweight: 41.89%. CIAF assessed more malnourished children than other approaches, including conventional indices. Our results and studies conducted in the Middle East and Africa seem consistent. Research in Nigeria’s rural community compared malnutrition through conventional indices and CIAF. Conventional indices were as follows: wasting at 14.1%, stunting at 33.1%, and underweight at 23.2%. At the same time, CIAF identified more malnourished children with multiple failures (overall 47.5%) [17]. A study in rural Yemen compared the nutritional status of children measured through CIAF and conventional indices. It verified that CIAF recognized more malnourished children (70.1%) compared to conventional indices in which 38.5%, 39.9%, and 55.1% stunting, wasting, and underweight prevalence [18].

When we compare our findings with the regional literature, we find that in India and Bangladesh, CIAF identified malnourished children greater than conventional indices or BMI and MUAC. In India, a study in rural Varanasi indicated that CIAF reported 62.5% of children malnourished compared to conventional indices (with percentages of 43.1, 35.2, and 31.5 incidences of stunting, under-weight, and wasting) [19]. Also, a study in Karnataka highlighted that around 58.4% of the children were screened for wasting, underweight, and stunting simultaneously using the CIAF tool. These rates are higher than conventional indices and MUAC at only 19.3 percent [20]. Another study in Haryana found that CIAF identified more malnourished children (45.25%) as compared to conventional indices (13.8% wasting, 31.2% stunting, and 21.4% underweight) [21]. Similarly, research in West Bengal highlighted that more children (32.7%) were reported malnourished through CIAF while through conventional method stunting was 15%, underweight and wasting was 17.7% [22]. Another study in West Bengal showed a higher prevalence of malnutrition through CIAF (61.6%) than other approaches such as MUAC (15.3%), BMI-for-age (13.4%), and conventional indices 51.9% stunting, 49.2% underweight, and 19% wasting [23]. Again in West Bengal, research showed CIAF was 36.1%, but stunting, wasting, and underweight prevalence were 4.9%, 2.1%, and 2.8%, respectively [24].

Next, a study in Kolkata revealed that the anthropometry approach identified 29% stunting, 30.5% underweight, and 28.8% wasting in under-five children, which was higher than the undernutrition reported through MUAC (20.3%) and BMI-for-age (28.8%) [25]. Also, an investigation in Gujarat revealed that the frequency of malnutrition through CIAF was 73.4 percent, whereas the rate of underweight through conventional indices was 50 percent [26]. A study in the slum areas of Chhattisgarh depicted that 62.1% of children identified as malnourished through the CIAF tool, while 45.2 %, 46.6 %, and 17.8% of children were screened as underweight, stunted, and wasted through conventional methods [27]. Another inquiry reported high malnutrition prevalence through MUAC (91.28% in boys and 88.55% in girls) compared to the anthropometric approach as stunting (39.74 percent in boys and 41.49 percent in girls) and wasting (19.55 percent in boys and 15.74 percent in girls) in Muslim population in West Bengal, India [28]. Another study compared the nutritional results in Delhi, India, measured through CIAF, MUAC, and conventional indices. It highlighted that CIAF screened 60.5 % malnutrition in children, which is higher than conventional indices in which stunting was 44.5%, underweight was 35.4%, wasting was 26.4%, and the MUAC noticed 23.7% of children malnourished [29].

The finding of the logistic regression models in Table 4 shows that all the variables are significantly associated with CIAF. The findings of the study revealed that the nutritious status of children is significantly impacted by age and gender. Some investigations depicted that age and sex largely influenced the child’s growth patterns [30]. A previous study in Guatemala showed that sex differences in length and weight existed over the age range as boys compared to girls were heavier and taller, and this difference was minimum at the birth of a child, but it increased over two years of age [31]. According to a study [32], female adults in South Asia and male adults in India, Bangladesh, Africa, and Pakistan were shorter in height than average in England. It depicted that differences in children’s height predominantly existed based on regional income, age, and sex differences. There is evidence that [33] in wealthy children, the sexual dimorphism normal pattern occurred when males had a propensity to be longer and heavier than females. The results of this study depicted that the probability of malnutrition prevalence is less in male children compared to female children in the district. Previous studies observed that due to gender inequality at the home level, i.e., differences in care methods during illness, and unequal distribution of food among male and female children, female children were more malnourished than male children [34,35,36].

The logistic results depicted that higher birth intervals decrease the chances of child malnourishment. On the other hand, it may not have a linear association with the rise in order of birth. It may be because the majority of the parents were satisfying the primary necessities of food for their children as food items such as vegetables, fruits, and milk are available at discounted rates and are accessible in rural regions. Research in Nepal revealed that the intermission of birth of fewer than 2 years was significantly correlated with severe acute malnourishment [37]. Similarly, an investigation of Pakistani culture demonstrated that higher birth order significantly raises the chances of stunting [38]. Although, another research in the Pakistani context revealed that the likelihood of child death reduced with higher intervals of birth [39].

It is considered that there may be a link between the women’s employment status and the care of a child. If the mother is involved in a proper type of job or work, then the child’s growth and care, particularly feeding of the child, might be influenced due to the less time provided to the child because of work or job [40]. Besides this, the women’s employment status contributes independently toward the nourishment of children. Family capital to purchase foodstuff and manage the fundamentals of life increases women’s earnings. The findings of this investigation demonstrated that mothers who were not employed have higher chances of malnutrition prevalence among their children. The findings of a research investigation in Pakistan depicted that the working status of women from the poorest and poorer households was not contributing to the nutritional status of children. In contrast, for women belonging to middle-income households, their working status contributed to the children’s nutritious status [41]. It can be determined that females’ work/job would be in a position that could uphold the family’s financial status, but the care of children should not be suffered or compromised. The study findings depicted that educated mothers have less likelihood of malnourishment prevalence among their children under the age of five. Certain past investigations also supported our findings that the education of females is a significant cause of malnutrition [42,43,44,45,46]. Another study from Sindh Province also identified maternal illiteracy, overcrowding, and low income as significant stunting risk factors [47]. Furthermore, education in rural mothers can reduce stunting, as women are more likely to be malnourished than men in these areas, meaning malnourishment prevalence in under-five children also increases [48].

The results depicted through logistic regression revealed that as the socioeconomic status increases, the chances of malnourishment prevalence in under-five children decrease. Research in India emphasized that household socioeconomic deprivation significantly influenced the nutrition status of children as a deficiency in basic facilities augmented the malnourishment occurrence in children [14]. Due to the lack of family socioeconomics in India, the greater part of the poor families had at least one weak or underweight child compared to non-poor families [49]. In Pakistan, a review of past studies showed that families with poor financial status were the foremost factor contributing to the malnourishment of the children [14,49,50,51,52,53,54,55,56,57]. By enhancing economic and social status, households have additional resources to afford food and nutrition for their children.

Around 38.9% of children’s z-scores in the study area were over the flag of WHO ranges (less than −5 and greater than +5), which depicts that one or two pieces of information, i.e., height, age, or weight, may be misinformed in data handling, or could be written/taken incorrectly. The chances of error in height and weight measurement were less. At the same time, it was noticed that most of the mothers in the study area were unsure of their children’s exact age, that perhaps they reported the wrong age. Because of this, 38.9% of children’s z-scores crossed the WHO ranges and skipped measuring the conventional indices and CIAF. Thus, in assessing malnutrition, care must be taken in anthropometric measurement that information on height, age, and weight of the child should be accurate. Especially age should be checked through children’s birth cards. 

Conventional indices covered malnutrition in only three groups of children (underweight only; wasted only; stunted only) while CIAF covered all groups of malnourished children [19]: underweight and wasted; underweight and stunted; stunted wasted and underweight; stunted and wasted. Thus CIAF seems a robust indicator for malnutrition assessment because it identified more children than conventional indices. It is developed from the combination of these three conventional indices. The additional dimensions of malnutrition unveiled by CIAF are its key worth and could have practical implications for policymakers to monitor the trend of malnutrition and needed resource allocation at a community level.

Limitation of the Study

This study has some limitations as it covers only one district of the Punjab province in Pakistan with a limited sample owing to financial and logistic limitations. These results have been based on the data collected from the poorest district of Pakistan. Generalizing these results could be difficult.

## 5. Conclusions

Malnutrition prevalence through MUAC, BMI-for-age, and conventional indices is lower than the CIAF approach. The results of binary logistic regression illustrated that all the explanatory variables have a significant association with CIAF compared to other anthropometric approaches, i.e., MUAC, BMI-for-age, and conventional indexes like stunting, wasting, and underweight. The study concludes that CIAF is a better tool for child nutritional assessment, which exhibits more accurate estimates of malnutrition. Also, it identifies more children having numerous anthropometric failures and provides malnourishment patterning among preschool children living in low-resource settings.

## Figures and Tables

**Table 1:** Sample size proportional distribution from sub-districts to UCs in the District.

District	Sub-Districts (Tehsils)	UC	~Sample from Each UC
Rahimyar Khan	Khanpur Sample size = 96	BaghoBahar	26
		Azeem Shah	34
		Kotla Pathan	36
	Liaquatpur Sample size = 81	Ghooka	25
		Shadani	26
		TrindaGurgaij	30
	Rahimyar Khan Sample size = 115	Bahishti	34
		Sonak	46
		Chak No. 84/P	35
	Sadiqabad Sample size = 92	Kot Sanger Khan	33
		Muhammad Pur	32
		Roshan Bhet	27
Total	Tehsils = 4	UCS = 12	384

**Table 2:** Children’s CIAF classification with anthropometric failure.

Indicators	Description	Stunting	Wasting	Underweight
A	No failure	No	No	No
B	Stunted only	Yes	No	No
C	Wasted only	No	Yes	No
D	Underweight only	No	No	Yes
E	Stunted and underweight	Yes	No	Yes
F	Wasted and Underweight	No	Yes	Yes
G	Stunted and wasted	Yes	Yes	No
H	Stunted, wasted & underweight	Yes	Yes	Yes

**Table 3:** Overall Malnutrition Prevalence (Frequencies and percentages) among children through different approaches.

Indicators	Frequencies and Percentages
BMI-for-age	Normal = 133 (25.73%); Undernutrition = 321 (62.24%); Overweight = 12 (2.32%); Obesity = 14(2.71%)
MUAC	Normal = 213 (44.68%); Moderate Undernutrition (MAM) = 113 (21.86%); Severe Undernutrition (SAM) = 173 (33.46%)
CIAF	Normal = 114 (36.77%) Malnourished = 196 (63.23%)
Stunting	Normal = 81 (30.34%); Moderate = 93 (34.83%); Severe = 93 (34.83%)
Underweight	Normal = 78 (29%); Moderate = 67 (24.91%); Severe = 124 (46.10%)
Wasting	Normal = 39 (54.93%); Moderate = 21 (29.58%); Severe = 11 (15.49%)

**Table 4:** Binary logistic regression analysis results for CIAF, MUAC, Child BMI-for-age, Conventional Indices, and their correlates.

Explanatory Variables	Categories	CIAF OR & CI	MUAC OR & CI	CBMI-for-Age OR & CI	Conventional Indices		
					Stunting OR & CI	Underweight OR & CI	Wasting OR & CI
Chid age (0 to 12 months-reference)	13–24 months	1.798 [0.65, 5.007]	0.68 [0.36, 1.27]	1.97 ** [1.07, 3.62]	2.09 [0.78, 5.57]	1.33 [0.67, 2.59]	1.27 [0.39, 4.18]
	25–36 months	6.33 *** [2.24, 17.90]	0.42 *** [0.23, 0.78]	2.14 ** [1.16, 3.94]	5.96 *** [2.36, 15.04]	1.12 [0.59, 2.11]	0.61 [0.18, 2.03]
	37–48 months	1.04 [0.45, 2.40]	0.26 *** [0.14, 0.48]	2.19 ** [1.19, 4.04]	1.22 [0.56, 2.69]	0.39 *** [0.21, 0.74]	0.72 [0.24, 2.17]
	49–60 months	0.84 [0.36, 1.97]	0.25 *** [0.33, 0.49]	2.81 *** [1.43, 5.54]	1.10 [0.49, 2.48]	0.23 *** [0.11, 0.47]	0.45 [0.12, 1.75]
Child gender (Female-reference)	Male	0.69 *** [0.41, 1.16]	0.89 [0.62, 1.31]	0.87 [0.59, 1.30]	0.67 [0.41, 1.10]	1.005 [0.67, 1.49]	1.38 [0.65, 2.92]
Birth order number (Birth order 1-reference)	2 or 3	0.90 [0.46, 1.77]	2.72*** [1.71, 4.34]	1.71 ** [1.07, 2.72]	0.61 [0.32, 1.16]	1.73 ** [1.04, 2.86]	4.09 ** [1.30, 12.84]
	4 or 5	0.51 ** [0.25, 1.01]	2.15*** [1.28, 3.62]	2.33 *** [1.34, 4.06]	0.52** [0.26, 1.004]	1.23 [0.68, 2.12]	2.10 [0.59, 7.54]
	6 and above	1.37 [0.54, 3.47]	3.68 *** [1.84, 7.35]	4.52 *** [1.87, 10.92]	0.87 [0.36, 2.06]	1.47 [0.73, 2.98]	2.99 [0.69, 12.88]
Mother’s education (Illiterate-reference)	Primary	0.002 *** [1.48, 5.48]	0.72 [0.34, 1.53]	0.61 [0.29, 1.25]	0.74 [0.36, 1.53]	1.01 [0.53, 1.94]	1.58 [0.55, 4.58]
	Middle	1.53 [0.44, 5.34]	0.77 [0.53, 2.33]	0.89 [0.36, 2.24]	1.24 [0.42, 3.69]	1.57 [0.73, 3.39]	2.42 [0.54, 10.88]
	Matric & higher	0.73 [0.19, 2.77]	0.71 [0.36, 2.03]	0.22 [0.44, 1.10]	1.004 [0.31, 3.24]	1.24 [0.24, 6.45]	1.57 [0.16, 15.92]
Mothers work Status (Working reference)	Not-working	8.06 ** [0.93, 69.93]	2.16 [0.67, 6.94]	1.91 [0.62, 5.84]	3.95 * [0.92, 16.91]	2.57 [0.74, 8.95]	1.69 [0.17, 17.23]
Household socio-economic status (SES-1-reference)	SES-2	0.002 ** [0.05, 0.88]	2.88 * [0.86, 9.65]	0.59 [0.17, 2.06]	0.42 [0.13, 1.38]	4.05 ** [1.21, 13.54]	10.89 *** [2.77, 42.83]
	SES-3	0.001 *** [0.01, 0.16]	1.52 [0.38, 6.14]	1.25 [0.27, 5.74]	0.25 ** [0.06, 1.04]	0.33 [0.08, 1.44]	1.54 [0.16, 15.18]
The overall significance of the models							
*: References: Odd Ratios; *p*-Values; Confidence Intervals Significance level: *** if *p* < 0.01 ** if *p* < 0.05, * if *p* < 0.1		Number of obs = 306	Number of obs = 517	Number of obs = 517	Number of obs = 312	Number of obs = 456	Number of obs = 407
		LR chi2(14) = 53.27	LR chi2(15) = 64.62	LR chi2(15) = 51.15	LR chi2(14) = 38.86	LR chi2(14) = 55.48	LR chi2(15) = 13.34
		Prob > chi2 ≤ 0.0001	Prob > chi2 ≤ 0.0001	Prob > chi2 ≤ 0.0001	Prob > chi2 = 0.0004	Prob > chi2 ≤ 0.0001	Prob > chi2 = 0.5764
		Pseudo R2 = 0.1318	Pseudo R2 = 0.0909	Pseudo R2 = 0.0802	Pseudo R2 = 0.0917	Pseudo R2 = 0.0895	Pseudo R2 = 0.0582

## Data Availability

The data used in this study are obtainable upon request from first author, due to ethical and privacy constraints.
